# Angle Dependence of Electrode Lead-Related Artifacts in Single- and Dual-Energy Cardiac ECG-Gated CT Scanning—A Phantom Study

**DOI:** 10.3390/jcm13133746

**Published:** 2024-06-27

**Authors:** Piotr Tarkowski, Elżbieta Siek, Grzegorz Staśkiewicz, Dennis K. Bielecki, Elżbieta Czekajska-Chehab

**Affiliations:** 1 Department of Radiology, Medical University of Lublin, 20-090 Lublin, Poland; elzbieta.czekajska-chehab@umlub.pl; 2 Department of Clinical and Radiological Anatomy, Medical University of Lublin, 20-090 Lublin, Poland; elzbieta.siek@umlub.pl (E.S.); grzegorz.staskiewicz@umlub.pl (G.S.); 3 Department of Radiology and Nuclear Medicine, University Hospital No 4, 20-090 Lublin, Poland; 4 Department of Diagnostic Imaging, Kings College Hospital, London SE5 9RS, UK; d.bielecki@nhs.net

**Keywords:** dual-energy CT, artifact reduction, ICDs

## Abstract

**Background**: The electrodes of implantable cardiac devices (ICDs) may cause significant problems in cardiac computed tomography (CT) because they are a source of artifacts that obscure surrounding structures and possible pathology. There are a few million patients currently with ICDs, and some of these patients will require cardiac imaging due to coronary artery disease or problems with ICDs. Modern CT scanners can reduce some of the metal artifacts because of MAR software, but in some vendors, it does not work with ECG gating. Introduced in 2008, dual-energy CT scanners can generate virtual monoenergetic images (VMIs), which are much less susceptible to metal artifacts than standard CT images. **Objective**: This study aimed to evaluate if dual-energy CT can reduce metal artifacts caused by ICD leads by using VMIs. The second objective was to determine how the angle between the electrode and the plane of imaging affects the severity of the artifacts in three planes of imaging. **Methods**: A 3D-printed model was constructed to obtain a 0–90-degree field at 5-degree intervals between the electrode and each of the planes: axial, coronal, and sagittal. This electrode was scanned in dual-energy and single-energy protocols. VMIs with an energy of 40–140 keV with 10 keV intervals were reconstructed. The length of the two most extended artifacts originating from the tip of the electrode and 2 cm above it—at the point where the thick metallic defibrillating portion of the electrode begins—was measured. **Results**: For the sagittal plane, these observations were similar for both points of the ICDs that were used as the reference location. VMIs with an energy over 80 keV produce images with fewer artifacts than similar images obtained in the single-energy scanning mode. **Conclusions**: Virtual monoenergetic imaging techniques may reduce streak artifacts arising from ICD electrodes and improve the quality of the image. Increasing the angle of the electrode as well as the imaging plane can reduce artifacts. The angle between the electrode and the beam of X-rays can be increased by tilting the gantry of the scanner or lifting the upper body of the patient.

## 1. Introduction

There are a few million patients with pacemakers (PMs) and implantable cardioverter-defibrillators (ICDs) worldwide, with approximately one hundred thousand more of these devices implanted yearly. Most of them are placed in the right ventricle (RV) and right atrium (RA). Electrical impulses from them are delivered into the patient’s heart via electrodes fixed in the heart wall. These are very reliable devices, but about 2–4% have lead-related problems yearly [1].

Due to the thin walls of the right heart chamber, stiffness of electrodes and lack of direct, visual control of the implantation process and perforation of the wall can occur, which may be a significant issue. Visualizing small and incomplete perforation is impossible to detect using conventional radiography or magnetic resonance imaging (MRI) and very difficult in echocardiography—only cardiac ECG-gated CT allows these defects to be visualized [2]. Also, artifacts related to the electrode, especially its tip, make this task challenging.

ECG gating can significantly reduce motion artifacts of the electrode in patients with low heart rates, but other types of artifacts such as photon starvation, blooming, and beam hardening cannot be avoided. These types of artifacts can be reduced by using dedicated software—metal artifact reduction algorithms (MAR), but some cannot be used in ECG-gated examinations. A relatively new technique is dual-energy CT, which has a lower susceptibility to beam-hardening artifacts. Another method is to increase kVp, which generates more high-energy photons less likely to be absorbed by the electrode but increases the radiation dose to the patient if others parameters remain unchanged.

Artifacts in CT imaging are any discrepancies between tissue attenuation on the CT image and the actual attenuation of the examined structure. These artifacts are significant problems in radiology because they can lead to false diagnoses and possible incorrect treatment. Technology to reduce them has been developed since the early days of CT scanners. Some types of artifacts are patient-related—movement and breathing—and can be avoided; others occur due to scanner malfunction—ring artifacts. Finally, some are created due to X-ray interaction with matter and rules of image reconstruction by CT scanners—beam-hardening, streak, and blooming artifacts. Dense objects like a focus of concentrated iodine contrast or metallic implants are a source of artifacts. They are problematic, especially in cardiac CT, because they can obscure delicate anatomical structures or resemble significant pathologies. They have been proven to mimic hypoperfusion areas or soft plaques [3].

Every experienced radiologist has observed that metallic objects like surgical screws or ICD electrodes placed at various angles to the scanning plane generate different artifacts, sometimes very clearly visible and other times barely noticeable. Sometimes, they render the entire image impossible to assess. This is an issue in CT and also CT-based attenuation correction for SPECT or PET/CT [4].

Technology to reduce artifacts in single-energy CT relies on iterative algorithms, sharper reconstruction filters [5], and increasing kVp. In the case of dual-energy CT, additional methods are available, like VMIs and material-specific images. The capability of dual-energy CT to reduce metal-related artifacts in non-cardiac studies has been reported for several years [6,7]. Nonetheless, there still needs to be more information about the application of these features in cardiac CT, because it is proven that ICDs lead can degrade image quality in coronary CTA [8]. A few phantom studies have been published about limiting metal artifacts in the cardiac dual-energy CT, but most are in-stent restenosis [9,10,11]. To our knowledge, the only paper regarding dual-energy CT and electrode-related artifacts was published by Reinartz et al. comparing the image quality of dual-source scanners examining mono- and dual-energy modes [12]. The most significant limitation of their study was scanning performed in just one plane of imaging (coronal) and using a subjective Likert scale.

This study aimed to compare the size of artifacts related to the tip of the ICD electrode and the defibrillating electrode imaged at different angles in relation to the imaging plane on images obtained in vendor-provided single- and dual-energy cardiac protocols using virtual monoenergetic images (VMIs).

## 2. Materials and Methods

All examinations were conducted with Revolution GSI CT Scanner (GE Healthcare, Waukesha, WI, USA). In this study, we used standard vendor-provided protocol used in our institution for standard-size patients. In order to maintain similar photon output in both protocols and therefor similar susceptibility to beam hardening, the vendor-provided single-energy protocol was set to manual mA instead of modulated. Technical parameters of single-energy and dual-energy are presented in table (Table 1).

### 2.1. Phantom

**Figure 1:**
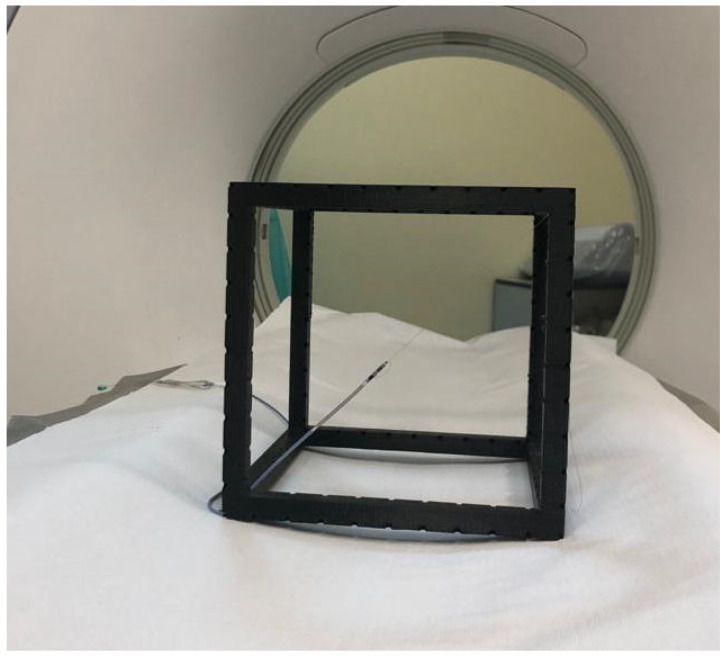
The frame with the electrode placed on the CT table.

The frame was printed using a 3D printer Creality ender 3, and each side was 20 cm high with holes every 1 cm in every bar to place the electrode (Figure 1), resulting in five-degree intervals. The electrode was attached to the frame using a thin fishing line invisible in CT scans.

### 2.2. The Course of the Experiment

The study commenced with scanning the electrode-free phantom to ensure that the material used did not introduce any artifacts. Subsequently, the electrode was affixed. A thin fishing line was attached to the coil used for electrode fixation, enabling stabilization of the electrode in the air away from the phantom walls to eliminate any artifacts.

The phantom with the set electrode at a 0-degree angle was positioned using a laser positioning system on the CT table to be perpendicular to the scanning plane and the long axis of the table. Holes in the frame walls used for threading the line and electrode served as reference points to ensure proper positioning. The electrode was then affixed as described above, and scanning was performed at a 0-degree angle in the examined plane. The electrode position was altered by threading the line through the next hole after each position change, with the correct phantom positioning confirmed using the positioning system. To achieve angles from 0 to 45 degrees, the opposite wall was utilized, while for angles from 50 to 90 degrees, the wall above the hole through which the electrode was passed was employed.

After obtaining scans in the sagittal plane, the same procedures were conducted for electrodes positioned in the coronal and axial planes. For the sagittal plane, the 0-degree angle positioned the electrode parallel to the long axis of the table. The same positioning was adopted for the coronal plane as a 0-degree angle. In the axial plane, the 0-degree angle positioned the electrode across the CT table, and the upward vertical position was the common position for the 90-degree angle in the sagittal plane.

Scanning was performed using a protocol consisting of two elements: first, dual-energy scanning and second, automatically triggered single-energy scanning. The single-energy scanning generated a series of images in the default window used in cardiology studies (WW/WL 800/100). The dual-energy scanning produced images with stored multi-energy data for later reconstruction, with the default value of VMIs set at 70 keV and the window configured the same as for SECT images.

**Figure 2:**
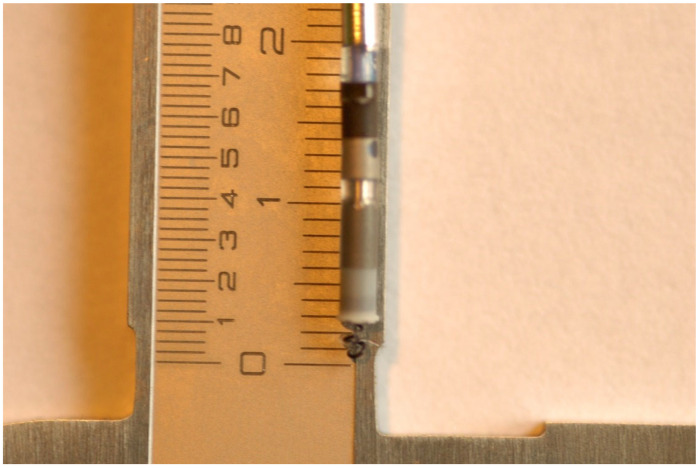
The measurements were performed at the end of the electrode—spiral attaching an electrode to the myocardium and 2 cm above it—corresponding to the beginning of defibrinating coil.

The study employed a defibrillating electrode type 10546687 IS-1BI manufactured by BIOTRONIK. The diameter of the electrode used was 2.52 mm–1/10 inch (Figure 2).

### 2.3. Measurements

All measurements were performed by two authors (E.S. and P.T.) using GSI Volume Viewer on GE AW 4.7 post-processing workstation (GE Healthcare, Waukesha, WI, USA) with window width and level 1200/200 on 40–140 keV virtual monoenergetic images (VMIs) with 10 keV interval reconstructed on the workstation. The length of the two most extended artifacts was measured on the original axial images at the end of the electrode and 2 cm from the point. These two points were chosen because it is essential to have a clear idea of the electrode’s tip to be able to exclude or confirm perforation. At 2 cm above the tip, very dense defibrillation part of ICD electrode starts, which generates a lot of artifacts.

### 2.4. Statistical Analysis

Statistical analysis was conducted using JASP 0.17.2 software (JASP Team 2023). The distribution type of variables was determined using the Shapiro–Wilk test. To compare variables with a normal distribution, the Student’s-t test was employed. For variables that did not exhibit a normal distribution, the Mann–Whitney test was utilized. Pearson correlation was used to determine the correlation between variables. Results were considered statistically significant when the significance level (*p*-value) was less than or equal to 0.05.

## 3. Results

**Chart 1:**
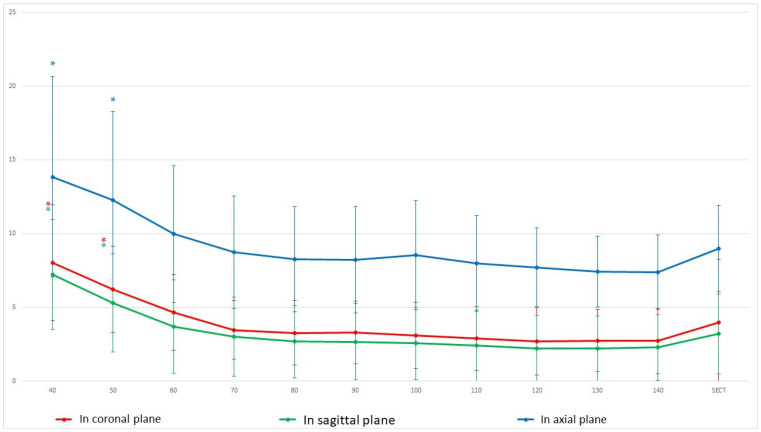
The size of streak artifacts originating from the electrode tip in VMIs and SECT images. Statistically significant differences compared to SECT are indicated by *.

**Chart 2:**
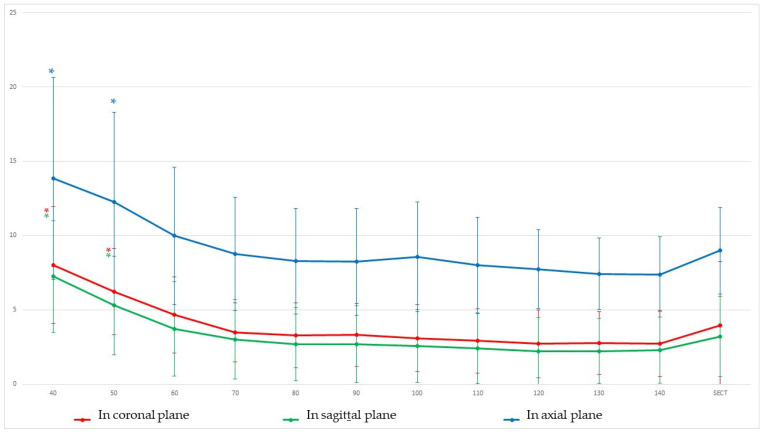
The size of streak artifacts originating from point 2 cm below tip of the electrode in VMIs and SECT images. Statistically significant differences compared to SECT are indicated by *.

The statistical analysis revealed that with an increase in VMI energy, streak artifacts originating from both the electrode tip and the defibrillator coils decrease (Chart 1, Table 2, Chart 2 and Table 3). VMIs with energy greater than 80 keV in each plane, for both measurement points, exhibited shorter artifacts compared to images obtained from SECT. However, the observed differences were not statistically significant. For both measurement points, the largest artifacts were observed when the electrode was in the axial plane—they were two to three times larger than in the other two planes, both in DECT and SECT examinations.

It was demonstrated that as the electrode approached the scanning plane in the sagittal (r = 0.855; *p* < 0.001) and frontal planes (r = 0.724; *p* < 0.001), the length of the artifacts originating from the defibrillator coil increased in the single-energy examination (Table 4). Similar observations were made for the electrode tip in both of these planes (r = 0.714; *p* < 0.001 and r = 0.815; *p* < 0.001, respectively) (Table 4).

Similar to the measurements conducted in the single-energy examination, it can be noted that as the electrodes approached the detector plane in the coronal and sagittal planes, an increase in the size of artifacts originating from the electrode tip was observed in all VMIs (Table 5).

When the electrode was in the axial plane, its inclination angle had no impact on the size of the artifacts originating from the tip (r = 0.091; *p* = 0.712) in the SECT images (Table 4). In the same images, it had a moderate impact on artifacts from the defibrillator coil (r = 0.532; *p* = 0.019) (Table 4).

In VMIs, there was a weak, positive correlation between the artifacts arising from the defibrillator coil and the angle in the axial plane (r = 0.328; *p* < 0.001). Unexpectedly, a weak, negative correlation was observed for the artifacts from the tip in VMIs (r = −0.322; *p* < 0.001) (Table 5).

Both in the examination conducted with the single-energy protocol and the dual-energy protocol, artifacts were statistically significantly larger when the electrode was in the axial plane compared to the other planes (*p* < 0.001) (Table 6). For the examination conducted with the single-energy protocol, there were no differences in the size of the artifacts from both measurement points positioned in the frontal and sagittal planes (*p* = 0.894—for the tip and *p* = 0.693 for the defibrillator coil) (Table 6). Also, monoenergetic images with the electrode in the transverse plane were burdened with the larger artifacts compared to the other planes (*p* < 0.001).

## 4. Discussion

The artifact problem in computed tomography (CT) has been an inherent problem since the inception of the first CT scans, and despite technological advancements, elimination of some artifacts remains problematic. Some artifacts stem from the interaction of X-ray radiation with matter and the manner in which CT images are reconstructed, making complete eradication improbable. Significant hopes for reducing metal artifacts are linked with dual-energy computed tomography (DECT). Over the past 15 years, numerous publications have demonstrated the feasibility of artifact reduction across various types of examinations using each of the dual-energy CT scanner models currently available on the market [13,14,15]. Most studies focused on orthopedics hardware and little attention was given to the problem of artifacts from metal in the cardiovascular system [16,17,18].

**Figure 3:**
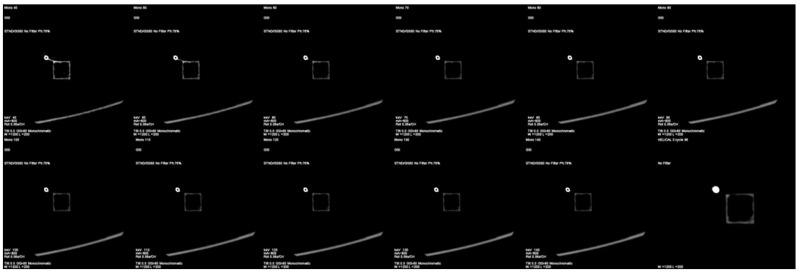
Series of virtual monoenergetic images (VMIs) 40–140 keV (from top left to right) when the electrode was placed in the sagittal plane. There is a slight reduction in artifact size, but they are generally very limited in this electrode position. The last one on the bottom right shows the polychromatic image.

**Figure 4:**
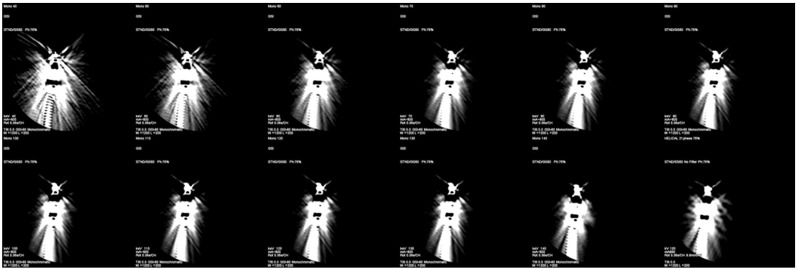
Series of monoenergetic images (VMI) 40–140 keV (from top left to right) when the electrode was placed in the axial plane at 90 degrees. The last one on the bottom right shows the polychromatic image. The VMI over 80 keV has fewer artifacts than the polychromatic image. There is a well-visible reduction in streak artifact length with increasing energy of the VMI.

**Figure 5:**
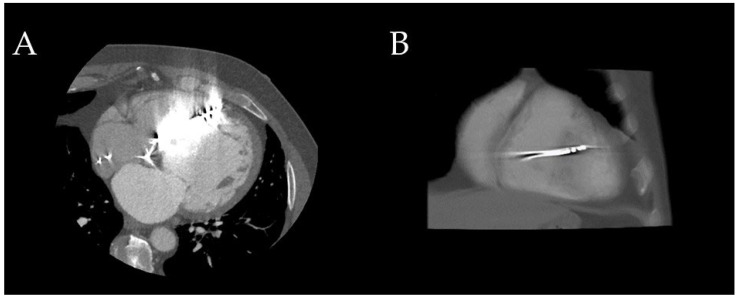
Clinical example of severe streak artifacts from ICD electrode positioned almost in axial plane. (**A**)—original SECT axial image WW/WL 800/100, slice thickness 0.625 mm, (**B**)—average slap image from coronal MPR demonstrating almost horizontal orientation of electrode.

Studies regarding the cardiovascular system were focused on coronary artery assessment. They have demonstrated that dual-energy computed tomography (DECT) using virtual monoenergetic images (VMIs) and material-specific images can improve the sensitivity and specificity of coronary artery stenosis evaluation by reducing artifacts from calcifications. These artifacts, which diminish the quality of the examination to a lesser extent than metals, are effectively mitigated through this approach [19,20]. There is only one study about artifacts from an ICD electrode [12]. It has been proven that artifact-related image deterioration is angle-dependent. This study has proven that the closer the electrode is to the imaging plane, the greater the observed artifacts (Table 3 and Table 4), which is clearly visible in Figure 3 and Figure 4. The severity of the artifacts arising from electrodes in clinical examination is demonstrated in Figure 5.

The relationship between the direction of the X-ray beam used in the examination and the location where the artifacts will be visible has long been recognized. This phenomenon has been utilized in several studies to partially displace artifacts away from the region of interest and reduce their severity, effectively improving the quality of the obtained images. These research findings align with previous reports indicating that the bigger the angle between the detector plane and the long axis of the prosthesis/electrode is to the X-ray beam, the smaller the observed artifact. This is due to the fact that the X-ray beam has to pass through a smaller thickness of metal, thus undergoing less hardening, which according to Beer’s law is proportional to the distance traveled [21,22].

## 5. Limitation of the Study

There was not a tissue-like structure around the electrode in the phantom, and it was surrounded by air, and for that reason, only hyperdense artifacts could be measured, which is a significant limitation of this study. Only half of the spectrum of the artifacts was measured for that reason.

Furthermore, there was no structure simulating the body of the patient and no adjustments to vendor-provided protocols were made to compensate for that fact, so with fixed mA for both DECT and SECT, there were more photons reaching the detector than in the examination with the human body, which may additionally reduce the artifacts.

Finally, no cardiac movement was simulated. Cardiac CT was performed during breath-hold, and for that reason, breath movement is not present, and its absence does not affect this study.

## 6. Conclusions

Virtual monoenergetic images can reduce streak artifacts arising from the tip and defibrillation part of the ICD electrode compared with single-energy CT. The length of the streak artifacts is angle-dependent. Some possible applications need further examination.

## Figures and Tables

**Table 1:** Acquisition parameters used in protocols.

Single-Energy Helical Cardiac CT Protocol	
Rotation time	0.28 s
Reconstruction filter	tandard
Slice thickness	0.625
Slice interval	0.625
kVp	120 kV
Current	constant 600 mA
Pitch	0.5:1
Dual-energy axial-cardiac CT protocol	
Rotation time	0.5 s
Reconstruction filter	standard
Slice thickness	0.625
Slice interval	0.625
kVp	80–140 kV
Current	constant 600 mA
Pitch	0.5:1

**Table 2:** The statistical significance of the difference in the size of streak artifacts between SECT images and VMIs at different energies in three planes and overall for the tip of electrode.

		SECT						VMIs					
			40	50	60	70	80	90	100	110	120	130	140
overall	The average length of artifacts [mm]	5.184	9.695	7.923	6.116	5.079	4.742	4.737	4.74	4.439	4.211	4.135	4.132
	*p*		<0.001	0.002	0.249	0.889	0.549	0.545	0.557	0.307	0.173	0.132	0.133
axial	The average length of artifacts [mm]	8.989	13.842	12.263	9.974	8.742	8.268	8.226	8.563	7.989	7.711	7.421	7.379
	*p*		0.007	0.04	0.439	0.824	0.499	0.477	0.695	0.323	0.167	0.79	0.077
sagittal	The average length of artifacts [mm]	3.353	8.011	6.216	4.663	3.479	3.274	3.305	3.089	2.905	2.705	2.758	2.732
	*p*		<0.001	0.012	0.214	0.897	0.937	0.962	0.793	0.654	0.523	0.548	0.536
coronal	The average length of artifacts [mm]	3.211	7.232	5.289	3.711	3.016	2.684	2.679	2.568	2.421	2.216	2.226	2.284
	*p*		<0.001	0.042	0.605	0.825	0.535	0.541	0.449	0.347	0.226	0.226	0.257

**Table 3:** The statistical significance of the difference in the size of streak artifacts between SECT images and VMIs at different energies in three planes and overall for the point 2 cm below tip of the electrode.

		SECT						VMIs					
			40	50	60	70	80	90	100	110	120	130	140
overall	The average length of artifacts [mm]	6.833	10.505	9.005	7.372	6.486	6.139	60.3	5.963	5.779	5.656	4.962	5.53
	*p*		0.002	0.053	0.612	0.733	0.486	0.417	0.387	0.288	0.236	0.227	0.185
axial	The average length of artifacts [mm]	13.353	18.395	16.732	14.558	13.179	12.547	12.321	12.579	12.142	12.026	11.974	11.7
	*p*		<0.001	<0.001	0.217	0.852	0.37	0.251	0.394	0.18	0.152	0.148	0.077
sagittal	The average length of artifacts [mm]	3.779	6.574	4.895	3.547	3.079	2.874	2.795	2.611	2.568	2.421	2.163	2.432
	*p*		0.011	0.278	0.812	0.471	0.355	0.316	0.221	0.205	0.151	0.17	0.16
coronal	The average length of artifacts [mm]	3.368	6.547	5.389	4.011	3.2	2.995	2.974	2.7	2.626	2.521	2.458	2.458
	*p*		0.004	0.058	0.463	0.823	0.615	0.588	0.355	0.305	0.238	0.199	0.209

**Table 4:** The correlation between the size of artifacts in SECT images and the angle of the electrode trajectory in three planes.

2 cm above Electrode Tip				
	Pearson r		*p*	
Sagittal plane	0.855	***	<0.001	
Coronal plane	0.724	***	<0.001	
Transverse plane	0.532	*	0.019	
Electrode Tip				
Sagittal plane	0.714	***	<0.001	
Coronal plane	0.815	***	<0.001	
Transverse plane	0.091		0.712	

* *p* < 0.05 and *** *p* < 0.001.

**Table 5:** The correlation between the size of artifacts in VMIs and the angle of the electrode trajectory in three planes.

2 cm above Electrode Tip				
	Pearson r		*p*	
Sagittal plane	0.648	***	<0.001	
Coronal plane	0.508	***	<0.001	
Transverse plane	0.328	***	<0.001	
Electrode tip				
Sagittal plane	0.242	***	<0.001	
Coronal plane	0.628	***	<0.001	
Transverse plane	−0.322	***	<0.001	

*** *p* < 0.001.

**Table 6:** Significance of difference length of the artifacts between different planes.

Planes	Tip	2 cm above the Tip
SECT		
Axial–sagittal	<0.001	<0.001
Axial–coronal	<0.001	<0.001
Coronal–sagittal	0.894	0.693
VMI		
Axial–sagittal	<0.001	<0.001
Axial–coronal	<0.001	<0.001
Coronal–sagittal	0.035	0.543

## Data Availability

Data are contained within the article.

## References

[1] Dereci A., Yap S.C., Schinkel A.F.L. (2019). Meta-Analysis of Clinical Outcome After Implantable Cardioverter-Defibrillator Implantation in Patients With Brugada Syndrome. JACC Clin. Electrophysiol..

[2] Rajkumar C.A., Claridge S., Jackson T., Behar J., Johnson J., Sohal M., Amraoui S., Nair A., Preston R., Gill J. (2017). Diagnosis and management of iatrogenic cardiac perforation caused by pacemaker and defibrillator leads. Europace.

[3] Levi J., Wu H., Eck B.L., Fahmi R., Vembar M., Dhanantwar A., Fares A., Bezerra H.G., Wilson D.L. (2021). Comparison of automated beam hardening correction (ABHC) algorithms for myocardial perfusion imaging using computed tomography. Med. Phys..

[4] Grosser O.S., Klutzny M., Wissel H., Kupitz D., Finger M., Schenke S., Wuestemann J., Lohmann C.H., Hoeschen C., Pech M. (2021). Quantitative imaging of bone remodeling in patients with a unicompartmental joint unloading knee implant (ATLAS Knee System)—Effect of metal artifacts on a SPECT-CT-based quantification. EJNMMI Phys..

[5] Bucher A.M., Wichmann J.L., Schoepf U.J., Wolla C.D., Canstein C., McQuiston A.D., Krazinski A.W., De Cecco C.N., Meinel F.G., Vogl T.J. (2016). Quantitative evaluation of beam-hardening artefact correction in dual-energy CT myocardial perfusion imaging. Eur. Radiol..

[6] Laukamp K.R., Große Hokamp N., Alabar O., Obmann V.C., Lennartz S., Zopfs D., Gilkeson R., Ramaiya N., Gupta A. (2020). Metal artifacts from sternal wires: Evaluation of virtual monoenergetic images from spectral-detector CT for artifact reduction. Clin. Imaging.

[7] Tarkowski P., Czekajska-Chehab E. (2021). Dual-Energy Heart CT: Beyond Better Angiography—Review. J. Clin. Med..

[8] Garmer M., Bonsels M., Metz F., Klein-Wiele O., Brandts B., Grönemeyer D. (2018). Coronary computed tomography angiography and endocardial leads-Image quality in 320-row CT using iterative reconstruction. Clin. Imaging.

[9] Bratke G., Hickethier T., Bar-Ness D., Bunck A.C., Maintz D., Pahn G., Coulon P., Si-Mohamed S., Douek P., Sigovan M. (2019). Spectral Photon-Counting Computed Tomography for Coronary Stent Imaging. Investig. Radiol..

[10] Kazimierczak W., Nowak E., Kazimierczak N., Jankowski T., Jankowska A., Serafin Z. (2023). The value of metal artifact reduction and iterative algorithms in dual energy CT angiography in patients after complex endovascular aortic aneurysm repair. Heliyon.

[11] Uliana Kay F. (2020). Dual-energy CT and Coronary Imaging. Cardiovasc. Diagn. Ther..

[12] Reinartz S.D., Kuhl C.K., Fehrenbacher K., Napp A. (2018). Magic Angle in Cardiac CT: Eliminating Clinically Relevant Metal Artifacts in Pacemaker Leads with a Lead-Tip/Gantry Angle of ≤70°. Acad. Radiol..

[13] Hickethier T., Baeßler B., Kroeger J.R., Doerner J., Pahn G., Maintz D., Michels G., Bunck A.C. (2017). Monoenergetic reconstructions for imaging of coronary artery stents using spectral detector CT: In-vitro experience and comparison to conventional images. J. Cardiovasc. Comput. Tomogr..

[14] Schwartz F.R., Tailor T., Gaca J.G., Kiefer T., Harrison K., Hughes G.C., Ramirez-Giraldo J.C., Marin D., Hurwitz L.M. (2020). Impact of dual energy cardiac CT for metal artefact reduction post aortic valve replacement. Eur. J. Radiol..

[15] Long Z., DeLone D.R., Kotsenas A.L., Lehman V.T., Nagelschneider A.A., Michalak G.J., Fletcher J.G., McCollough C.H., Yu L. (2019). Clinical assessment of metal artifact reduction methods in dual-energy CT examinations of instrumented spines. Am. J. Roentgenol..

[16] D’Angelo T., Cicero G., Mazziotti S., Ascenti G., Albrecht M.H., Martin S.S., Othman A.E., Vogl T.J., Wichmann J.L. (2019). Dual energy computed tomography virtual monoenergetic imaging: Technique and clinical applications. Br. J. Radiol..

[17] Katsura M., Sato J., Akahane M., Kunimatsu A., Abe O. (2018). Current and novel techniques for metal artifact reduction at CT: Practical guide for radiologists. Radiographics.

[18] Byl A., Klein L., Sawall S., Heinze S., Schlemmer H.P., Kachelrieß M. (2021). Photon-counting normalized metal artifact reduction (NMAR) in diagnostic CT. Med. Phys..

[19] Gupta A., Kikano E.G., Bera K., Baruah D., Saboo S.S., Lennartz S., Hokamp N.G., Gholamrezanezhad A., Gilkeson R.C., Laukamp K.R. (2021). Dual energy imaging in cardiothoracic pathologies: A primer for radiologists and clinicians. Eur. J. Radiol. Open.

[20] Yunaga H., Ohta Y., Kaetsu Y., Kitao S., Watanabe T., Furuse Y., Yamamoto K., Ogawa T. (2017). Diagnostic performance of calcification-suppressed coronary CT angiography using rapid kilovolt-switching dual-energy CT. Eur. Radiol..

[21] Osman N.D., Suhaimi N.M., Saidun H.A., Razali M.A.S.M., Sobri N.F.M. (2019). Metal artefact reduction with different transverse angles of metal placement and gantry tilt angulation in spine CT imaging. Malaysian J. Med. Health Sci..

[22] Marcus R.P., Morris J.M., Matsumoto J.M., Alexander A.E., Halaweish A.F., Kelly J.A., Fletcher J.G., McCollough C.H., Leng S. (2017). Implementation of iterative metal artifact reduction in the pre-planning-procedure of three-dimensional physical modeling. 3D Print. Med..

